# Precision Assembly of Complex Cellular Microenvironments using Holographic Optical Tweezers

**DOI:** 10.1038/srep08577

**Published:** 2015-02-26

**Authors:** Glen R. Kirkham, Emily Britchford, Thomas Upton, James Ware, Graham M. Gibson, Yannick Devaud, Martin Ehrbar, Miles Padgett, Stephanie Allen, Lee D. Buttery, Kevin Shakesheff

**Affiliations:** 1 School of Pharmacy, University of Nottingham, Nottingham, NG7 2RD, UK; 2 School of Physics and Astronomy, University of Glasgow, Glasgow, G12 8QQ, UK; 3 Department of Obstetrics, University Hospital Zurich, 8091 Zurich, Switzerland

## Abstract

The accurate study of cellular microenvironments is limited by the lack of technologies that can manipulate cells in 3D at a sufficiently small length scale. The ability to build and manipulate multicellular microscopic structures will facilitate a more detailed understanding of cellular function in fields such as developmental and stem cell biology. We present a holographic optical tweezers based technology to accurately generate bespoke cellular micro-architectures. Using embryonic stem cells, 3D structures of varying geometries were created and stabilized using hydrogels and cell-cell adhesion methods. Control of chemical microenvironments was achieved by the temporal release of specific factors from polymer microparticles positioned within these constructs. Complex co-culture micro-environmental analogues were also generated to reproduce structures found within adult stem cell niches. The application of holographic optical tweezers-based micromanipulation will enable novel insights into biological microenvironments by allowing researchers to form complex architectures with sub-micron precision of cells, matrices and molecules.

The study of multicellular microenvironments is challenging due to the lack of effective technologies to recreate cellular architectures at the micron scale. The generation of more sophisticated tools to study these structures will have applications in numerous areas including basic biochemical and biomedical research, regenerative medicine, tissue engineering, biophysics and many others.

The precise architectural position of cells within a particular microenvironment provides the basis for biological function. This is exemplified in early embryogenesis where the organization of less than 16 individual blastomeres controls subsequent development into the morula and blastula^1^. Any technique designed to rebuild such structures must therefore have the capacity to position at a resolution lower than that of an individual cell which varies depending on cell type and stage of development; for example human blastomeres have a diameter of ~80 μm at the two-cell stage and ~65 μm at the four-cell stage^1^. The lack of non-destructive methods of micromanipulation has therefore limited functional determinations to observational and molecular biological approaches.

The structures present within adult organisms are also spatially organized at small length scales, for example within stem cell niches^2^,^3^. Regulation of stem cell activity within these structures results from the interplay between the intrinsic genetic regulatory pathways of the stem cells themselves^2^ and positional associations with soluble factors, extracellular matrix (ECM) interactions, cell-cell interactions and mechanical stimulation^3^.

Early work to determine the structure and function of these interactions used genetic models such as *Caenorhabditis elegans*^4^,^5^ and *Drosophila*^6^,^7^ which in turn provided insight into mammalian systems^8^ due to the evolutionary conserved nature of these structures. Studies involving mammalian cultures have primarily involved the use of two dimensional co-culture experiments and tissue explants^9^,^10^,^11^. The use of explants is further limited by physiological position with more accessible structures such as the bulge area of the hair follicle being easier to dissect for *in vitro* culture^10^,^11^. *In vitro* screening techniques such as robotic spotting^12^,^13^, the generation of microfabrication wells^14^,^15^ and bioprinting applications have generated spatially orientated co-cultures^13^,^16^.

Whilst these techniques have provided invaluable information on regulatory pathways they illustrate the current inability to determine the microscopic structure to function relationships between different cells as well as other micro-environmental components. Holographic optical tweezers (HOTs) are a micromanipulation tool of sufficient resolution to study these properties due to their capacity to position microscopic objects such as cells accurately and in three dimensions (3D)^17^,^18^,^19^,^20^,^21^,^22^,^23^,^24^.

Applications of optical tweezers technology have to date focused on determining the biophysical properties of cells^25^,^26^ but have also been used to position biological structures including plant cells^19^, bacteria^20^, yeast^27^ and mammalian cells such as retinal neurons^17^, B cells^21^ and stem cells^22^,^24^,^28^. Whilst such applications demonstrated the principle of biological trapping, these studies were limited in their ability to generate defined cellular architectures in 3D and retain cultures for further biological analysis^17^,^21^,^22^. Accurate recreation of complex cellular structures such as the stem cell niche also requires the control of the physical and chemical properties of the surrounding microenvironment.

We demonstrate the combination of a HOTs system with controllable and tailored structural elements including polymeric materials, ECM, controlled release microparticles and hydrogels. These elements were micro-manipulated into complex architectures precisely controlling physical and chemical factors to produce micro-environmental analogues. This powerful new tool will enable the study of regulatory mechanisms in diverse cellular microenvironments producing novel insights at a scale and level of micro-architectural complexity that previous techniques have been unable to provide.

## Results

### Generating cellular architectures using HOTs

**Figure 1:**
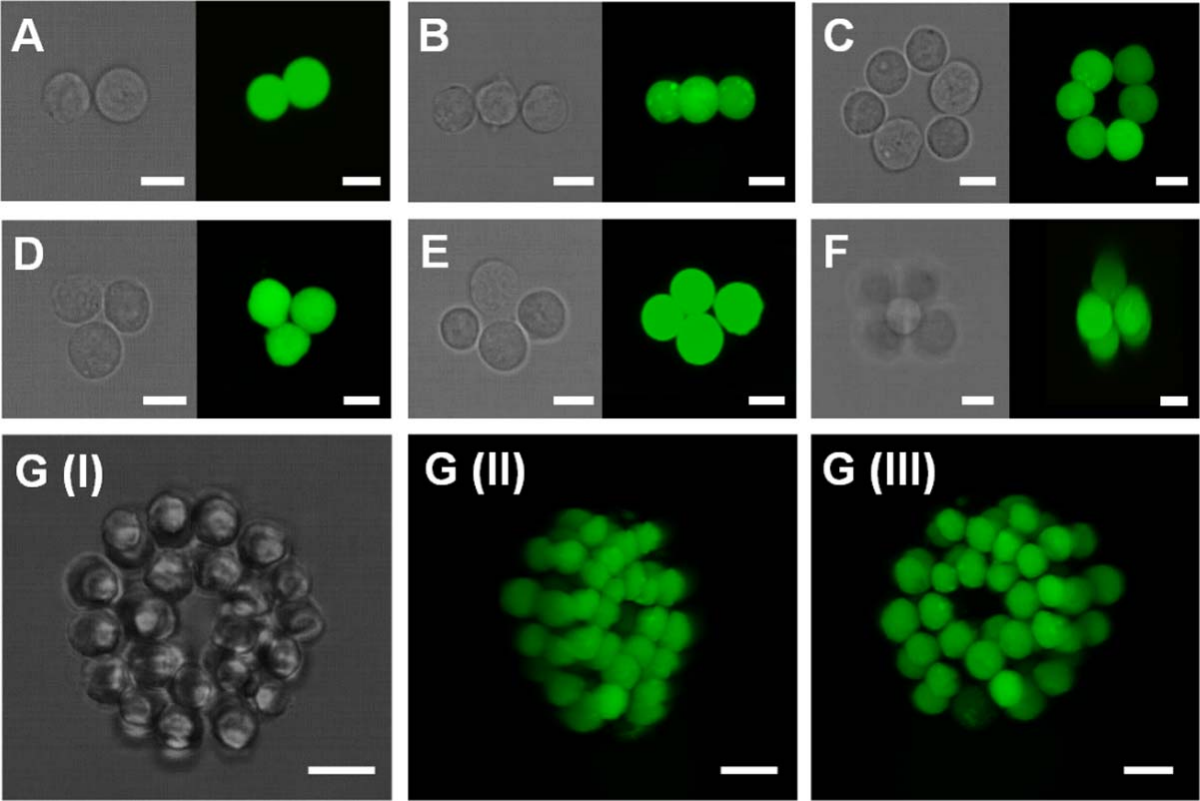
Images of mouse embryonic stem cells patterned into precise 3D structures using holographic optical tweezers with resulting architectures stabilized using either an avidin-biotin method of cell adhesion (A–F) or a PEG based modular hydrogel matrix (G). Images were taken in brightfield and using fluorescent confocal microscopy (green labelled images). Scale bars = 12 μm.

To enable the HOTs system (Supplementary Fig. 1) to position small groups of cells into precise positions a bespoke click-and-drag based software interface was programed which induced real-time changes in the hologram configuration facilitating the movement of optically trappable elements in real-time and in 3D (Supplementary Movie 1). To test this capacity we successfully patterned cellular architectures of mouse embryonic stem (mES) cells of up to six in number generating cellular structures positioned within the visible plane. The resulting architectures were found to be highly unstable with rapid reorganization of cellular orientations causing a loss of positional accuracy. To retain cellular position we used a rapid cell crosslinking strategy that forces cell-to-cell contact for the first 24 hours in culture. Aldehyde groups were generated on the surface of the mES by use of sodium periodate facilitating the binding of biotin hydrazide^29^. These cells were then pattered within medium containing avidin and the resulting cross-linking mechanism used to stabilize the patterned structure formed (Fig. 1a–e).

Having established the capability of this technique to generate two dimensional structures we then moved on to investigate 3D patterning by positioning mES into simple, low cell number architectures (Fig. 1f). The positioning of larger cell number structures in 3D (Fig. 1g) was achieved by the integrated use of both the microscope focus drive and the induction of phase shifts within the hologram which generated corresponding changes in the z position of specific, individual traps within the plane of focus. The combined use of these produced two levels of z-axis control, coarse control produced by the focus drive and fine control from the digital hologram configuration, enabled accurate positional control in all three dimensions.

Using this capacity we generated larger cellular structures by initially positioning a ring of eight cells around which we placed 14 mES cells forming a second ring that surrounded the initial structure with the cells on the outer and inner rings forming avidin-biotin adhesions. This unit was further expanded by the addition of two cellular layers in identical configurations making a multi-layer, integrated, stable, ring based structure of 66 cells (Fig. 1g and Supplementary Movie 2). In this instance the larger structure required additional support after patterning than the avidin-biotin method could provide.

To retain architectures indefinitely within larger structures and cultures where elements were placed at defined distances away from other objects such as mES cells a physical, stabilizing medium was introduced in the form of hydrogels. The properties of this surrounding medium also introduced an additional level of biophysical control within the constructed microenvironment. This was achieved by use of a thermoresponsive gel system ULMP agarose and a polyethylene glycol (PEG) based modular system (Fig. 1g, 3a, 4).

The PEG based modular gel matrix consisted of two multiarm PEG-peptide conjugates that were functionalized to enable cross-linking by the addition of the transglutaminase factor XIIIa^30^. The gel was formulated with 1.5% (w/v) PEG precursors, which contained a matrix metalloproteinase (MMP) cleavable linker facilitating the integral cross-linking of L-Arginyl-Glycyl-L-Aspartic acid (RGD). This system was used to stabilize larger scale mES structures (Fig. 1g) as well as to fix the position of smaller architectures within the gel for subsequent analyses. The physical properties of this material can be tailored to the desired application and a wide range of proteins and peptides can be integrated within the matrix^31^,^32^.

**Figure 2:**
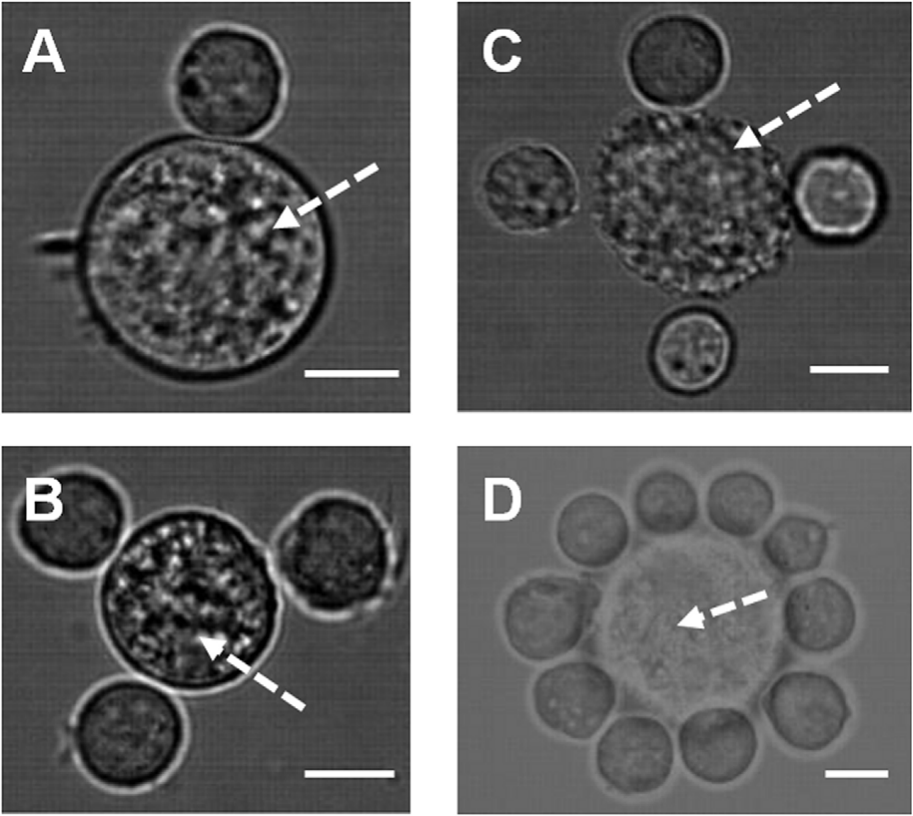
Patterning of multiple cell types using holographic optical tweezers in co-cultures of: (A–B). Mouse embryonic and mesenchymal (arrow) stem cells. (C–D). Mouse primary calvarae cells (arrow) and embryonic stem cells. Imaging was achieved by use of integrated fluorescent and bright-field imaging system. Scale bar = 12 μm.

Having established the ability of this technique to generate architectures using a single cell type, we next built structures in which the location of multiple cell types could be independently controlled. We created patterns within co-cultures by positioning mES cells and mouse mesenchymal stem cells (mMSCs) into defined architectures (Fig. 2a, b) as well as mES and mouse primary calvarea (mPC) cells (Fig. 2c, d). In order to distinguish between differing cell types during the patterning process fluorescent cell tracker probes were used to label both mMSCs and mPC but not mES. The visualization of these cells was achieved by building fluorescence microscopy capacity into the system capable of holding filter cube sets of defined wavelengths and used in combination with appropriate light emitting diode sources. The system is capable of visualizing multiple fluorescent probes and could therefore be adapted to pattern numerous distinct cell types simultaneously.

### Simultaneous positioning of cells and polymer structures

**Figure 3:**
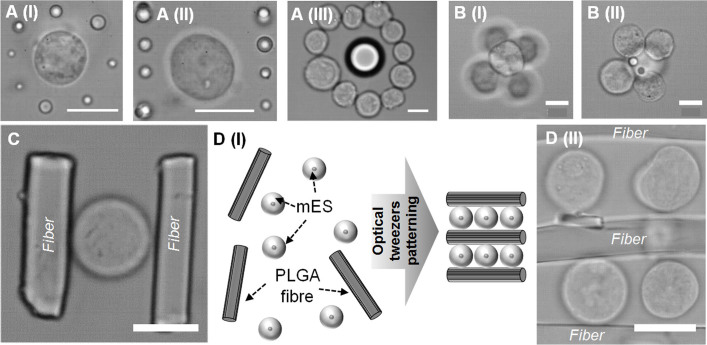
(A). Precision patterning of mouse embryonic stem cells and PLGA microparticles of varying sizes using holographic optical tweezers. (B). PLGA microparticles patterned within mES aggregates. (C). Precision patterning of mouse embryonic stem cells and fibronectin coated poly (D, L-lactide-co-glycolide) electrospun fibers. (D). Patterning of fibers and mES into layered structures. Scale bars = 12 μm.

The positioning of multiple cell types into small (<10) and larger scale structures using this technology provides a tool for the study of many *in vivo* microenvironments. However, the technique can create more complex microenvironments by simultaneously patterning both cells and materials. To demonstrate this concept, polymer materials were positioned within cell cultures in the form of microparticles and electrospun fibers. We propose this as a means to control physical environments, tailoring materials and their surface properties to the required application. Poly(DL-lactic-co-glycolic acid) (PLGA) microparticles and electrospun fiber fragments were trapped and positioned within mES cell cultures forming predefined structures (Fig. 3). PLGA microparticles of various sizes were trapped and positioned in 3D at predefined positions and distances from the edge of individual and groups of mES cells (Fig. 3a). These microparticles were also positioned within cellular structures as they were constructed on the tweezers system (Fig. 3b) and the resulting patterns stabilized by use of either an ultra-low melting point (ULMP) agarose hydrogel (Fig. 3a) or the avidin-biotin method (Fig. 3b).

To further expand the range of material structures that could be used in micro-environmental construction, PLGA microfibers were generated by electrospinning, cryogenically ground and fractioned according to size. The microfibers were then treated with fibronectin to facilitate cell adhesion; since neither the avidin-biotin nor hydrogel systems were used for the microfiber patterning. These cell adhesive fibers were suspended within mES cell cultures to generate cell and material co-patterns. Simple structures, for example consisting of one cell and two small PLGA fiber fragments were easily be constructed (Fig. 3c) as well as larger architectures consisting of parallel lines of cells sandwiched between individual fiber fragments (Fig. 3d). All microfiber manipulations were performed using the 100 × microscope objective as stable trapping could not be achieved at lower magnifications.

### Temporal control of the chemical environment

**Figure 4:**
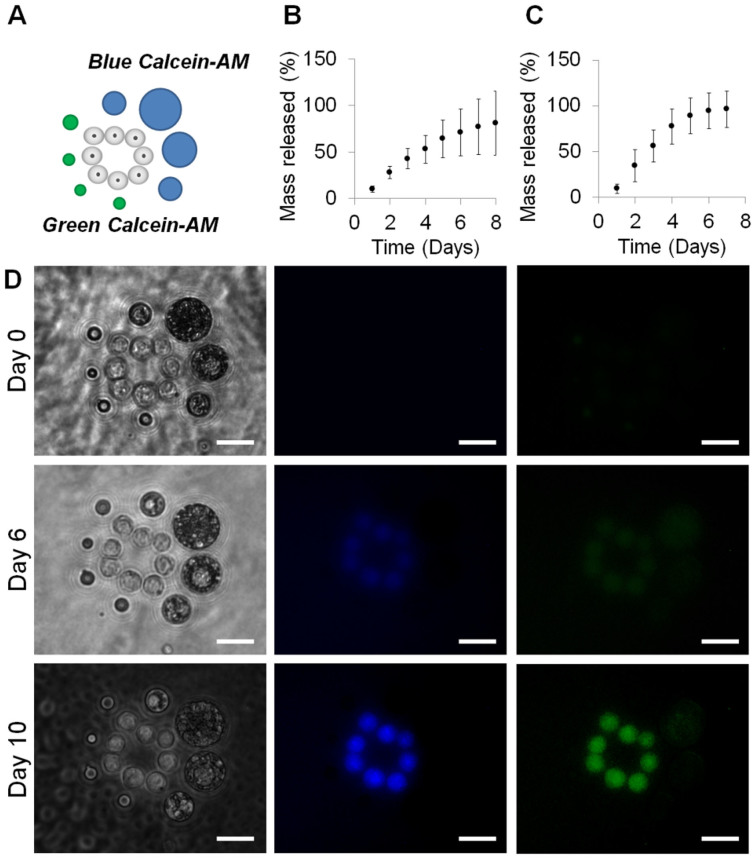
Controlled release of green and blue labelled Calcein-AM from PLGA microspheres positioned at precise locations around mES cells within agarose gels and cultured over 10 days. (B). Release profiles for blue Calcein-AM microparticles. (C). Release profiles for green Calcein-AM microparticles. (D). Bright-field and fluorescent images of resulting cultures at zero, six and 10 days after pattern formation. Scale bars = 12 μm.

This technology was also designed to introduce temporal control elements within the patterned structures that could release chemical factors which form part of specific cellular microenvironments. We utilized established polymer microparticle based controlled release technology with specific formulations tailored to produce desired release profiles (Fig. 4a)^33^. To visually demonstrate this principle two batches of PLGA microparticles were formulated to release either blue (Fig. 4b) or green (Fig. 4c) Calcein-AM over a 10 day period. In order to visually distinguish between the green and blue loaded Calcein-AM particles during patterning each was formulated to have distinct size ranges with larger for blue loaded particles (19 μm average diameter) and smaller for green (8 μm average diameter) (see Supplementary Fig. 2).

Each microparticle formulation was positioned on one side of a ring of mES cells as shown in the schematic (Fig. 4a). The position of these elements was stabilized by use of a ULMP agarose hydrogel system and left in culture for 10 days. Calcein-AM is a non-fluorescent acetomethoxy derivate of calcein that can be transported across the cell membrane into living cells where intracellular esterases cleave the acetomethoxy group hence this molecule will only become fluorescent intracellularly. The cells began to show low level fluorescence in both the green and blue spectra at six days, reaching maximum fluorescence levels at 10 days after patterning (Fig. 4d). The use of a viability stain in this manner also confirmed the viability of the cells in culture after the 10 day period.

### Adult stem cell co-culture system

**Figure 5:**
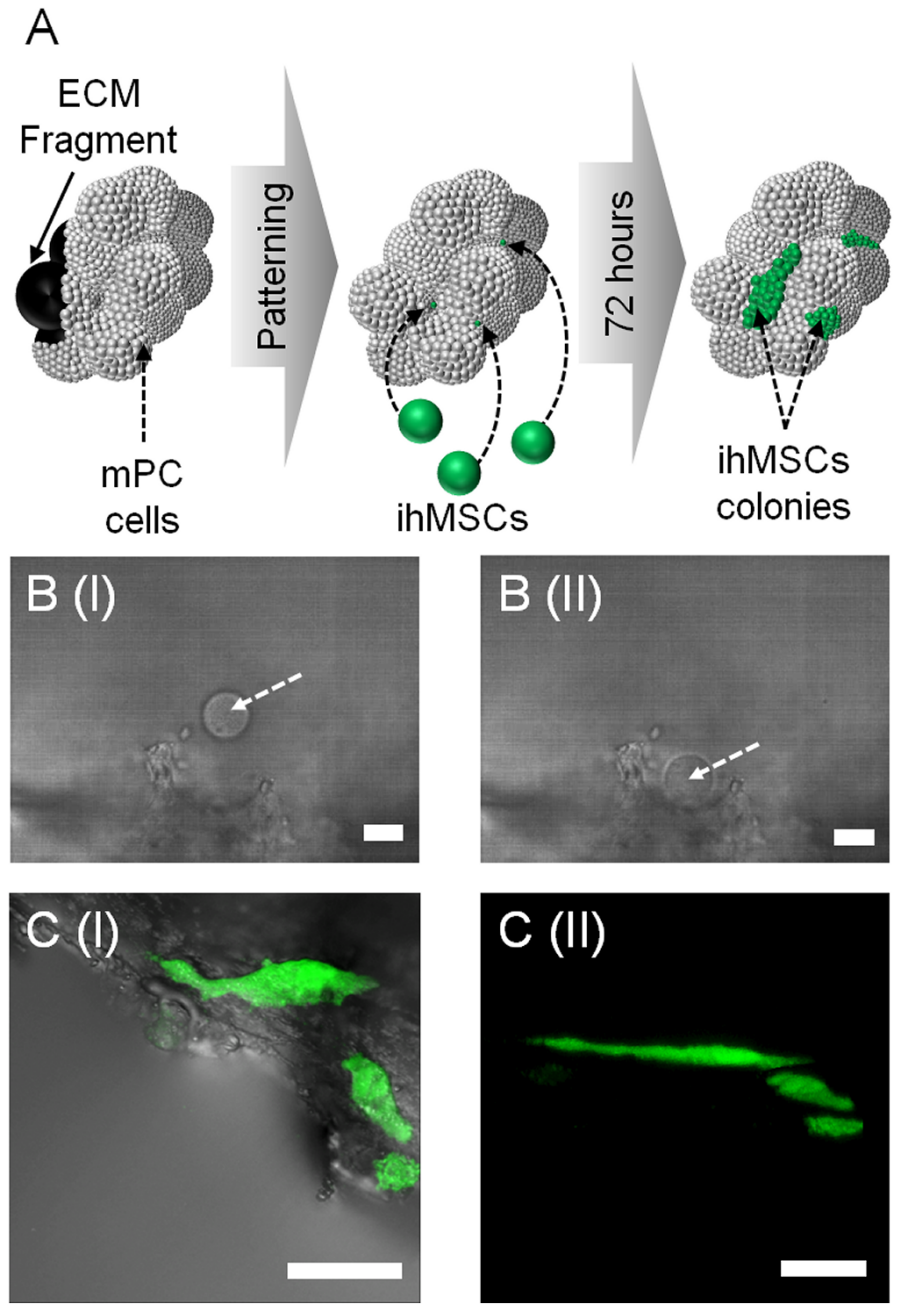
A simplified *in vitro* model of an adult stem cell co-culture system. (A). A diagrammatic representation of demineralized and decellularized ECM fragments re-seeded with mouse primary calvarae cells onto which individual ihMSCs were seeded into precise positions within the porous structure and left in standard culture conditions for 72 hours. Scale bars = 12 μm. (B). A brightfield image of immortalized human mesenchymal stem cells (ihMSCS) (indicated with an arrow) before (I) and after (II) seeding of ihMSCs. Scale bars = 12 μm. (C). (I) Merged transmission and fluorescent macroconfocal image of resulting cultures after 72 hours. (II) 3D reconstruction using macroconfocal imaging of 72 hour culture. Scale bars = 450 μm.

The data thus far demonstrates the potential applications of this method of HOT manipulation in a wide range of applications. To further develop this technique we also investigated the capacity to construct more complex micro-environmental structures. We generated acid demineralized and enzymatically decellularized ECM fragments from mouse trabecular bone samples forming a bioactive scaffold^34^. These fragments were then re-cellularized with mPC cells forming the base for the HOTs based patterning of specific cell types (Fig. 5a).

We investigated the capacity of this construct to support the clonal expansion of individually seeded adult stem cells. To assist in the visualization of such colonies and to ensure continuous proliferation an immortalized cell line of human MSC (ihMSCs) origin was used with an integrated green fluorescent protein (GFP) reporter. Individual mPC cellularized ECM fragments were loaded into microwells for patterning on the tweezers micromanipulation system and a very dilute cell number suspension of ihMSCs was then added to each well. Single ihMSCs were placed in isolation within the porous structure of the re-cellularized ECM (Fig. 5b) and the position noted by use of positional tracking capacity of the software relative to a pre-selected visual reference point. The cultures were then returned to standard culture conditions and left for 72 hours to allow for expansion of the cells into colonies. The resulting cell colonies were visualized using macroconfocal imaging and showed growth into distinct, expanded colonies within the regions of initial seeding (Fig. 5c).

### Assessment of cell death due to optical trapping

The data discussed in this paper demonstrates the potential of this HOTs based biological manipulation technology but any detrimental effects are an important consideration. The damaging properties of infrared (IR) laser radiation are limited to heat induction^35^,^36^. Water is the primary constituent of mammalian cells (~70%) and has a low absorption at 1064 nm inducing 1.15 K heating for every 100 mW of laser energy^37^. The calculated laser energy of each trap on our system does not exceed 30 mW which would induce an increase of less than 0.35 K and only within the localized trapping area.

We also investigated if the mechanical effects of the optical trapping had negative effects on viability. To do this cells were positioned into six cell ring structures and exposed to 10, 20 or 30 minutes of optical trapping (a single trap per cell) (Supplementary Fig. 3a). There was no increase in the number of dead cells in cultures exposed to these trapping exposure procedures compared to ring structures that were formed but were not trapped further (Supplementary Fig. 3b), as well as to cultures where cells had not been exposed to any form of optical trapping (Supplementary Fig. 3c). All cellular positions, both patterned and non-patterned were stabilized by use of a PEG hydrogel system to ensure positional and pattern stability.

## Discussion

The biological microenvironments within developing embryos and adult organisms consist of highly complex arrangements of cells, ECM and chemical factors^9^,^10^,^11^. The structural organization of these elements and mechanisms that regulate them form the basis for biological function. The recreation of these structures *in vitro* has to date been challenging due to a lack of technologies that can position individual cells and matrices at a sufficiently small length-scale.

Techniques such as robotic spotting^12^,^13^, the generation of microfabrication wells^14^,^15^, two dimensional micropatterning^38^ and bioprinting applications^13^,^16^ can generate spatially orientated co-cultures but are unable to position individual cells and materials at sufficiently high resolution and at the required level of architectural complexity. Furthermore, any method able to achieve this must also have the capacity to arrange multiple elements simultaneously within co-culture systems.

HOTs^17^,^18^,^19^,^20^,^21^,^22^ can manipulate individual^28^ as well as groups of cells^28^ in 3D and their positional resolution can be used not only to precisely manipulate cells but also subcellular components^26^. Ma and colleagues positioned single MSCs onto cell monolayers and monitored subsequent cell responses^24^ while Akselrod *et al* generated heterotypic microarrays in which mammalian and bacterial cells were positioned within co-cultures^23^. While these and other such studies illustrate the principle of biological patterning they lack a sufficient level of 3D architectural organization to truly recreate *in vivo* microenvironments.

In order to realize the full potential of HOTs we positioned differing cell types, polymeric materials, ECM and controlled release technology into precisely defined 3D architectures (Fig. 1,2,3,4,5). It is this precise combination of technologies that has enabled the generation of multifaceted micro-environmental analogues with a level of complexity that, to our knowledge, has not previously been demonstrated.

The retention of micro-structural organization after HOTs patterning was achieved by use of either avidin-biotin chemical adhesion (Fig. 1, 2) or hydrogels (Fig. 1g, 4). Previous studies have used hydrogels as a post-patterning stabilization medium^18^,^23^,^24^ but have not utilized these technologies as a means of micro-environmental control. We used a modular PEG based hydrogel system modified to contain the cell adhesion moiety RGD (Fig. 1g, Supplementary Fig. 1) to illustrate this capacity. The flexibility of this hydrogel allows the incorporation of a wide range of bioactive molecules including growth factors and cytokines^30^,^31^,^32^. Immobilization of specific cues in this manner both controls the stiffness and density of the hydrogel and provides additional means of tailoring micro-environmental properties.

Diverse hydrogel technologies are currently being developed, generating ever more sophisticated analogues of ECM function for application in the study of micro-cellular activities^39^. For example, a study by Lou *et al* demonstrated biomolecule incorporation into defined channels (<200 μm diameter) within a bulk ULMP agarose structure which induced outgrowth of rat dorsal root ganglia^40^. Although such techniques are generating significant new insights they are unable to fully assess how the precise architectural organization of individual cells determines micro-environmental activities. The use of our HOTs method with such hydrogel systems will therefore greatly enhance their ability to study such mechanisms.

While the precision control of cellular organization and hydrogel properties provides an invaluable micromanipulation tool-set, the control of the soluble factors is of equal importance. We used polymer microparticle based controlled release technology^33^ to position sources of Calcein-AM release within structures during HOTs patterning providing a visual proof-of-principle (Fig. 4). We have also previously demonstrated the temporal release of growth factors and cytokines from microparticles^33^. The use of such formulations will allow the tailored delivery of specific signaling elements within biologically relevant *in vitro* models mimicking, for example, the microenvironments present within developmental biology.

The complex patterning of cells in isolation will provide a tool within fields such as developmental biology (Fig. 1, 2, 4) but is insufficient for the construction of microenvironments found within hard tissues such as bone. To address this we developed polymer structures in the form of microparticles, fragments of ECM and PLGA electrospun fibers which we positioned within cell cultures during HOTs pattering (Fig. 3, 5). The properties of these materials can also be tailored to a specific microenvironment due to their diverse properties and applications with biomedical research.

The application of our method of HOTs based micro-manipulation will enable micro-environmental characterization at a scale and resolution that to our knowledge has not previously been demonstrated. The key advantages of this technique are the ability to generate cellular constructs in 3D, introduce controllable and tailored structural elements at precise positions during construction and the temporal control of chemical factors. The systems we have demonstrated can be applied to a wide range of applications including developmental biology and the study of *in vitro* microenvironments such as those found with stem cell niches, enabling new insights that other technologies are unable to provide.

## Methods

### HOTs micro-manipulation system

The HOTs instrument was similar to a system detailed previously^41^ and was based on an infra-red laser for bio applications; Supplementary Fig. 1 shows a schematic representation of the system. All software based control functions were programed using Labview software (National Instruments) as described previously^42^,^43^. A 40 × objective with a numerical aperture (NA) of 1.3 (Zeiss) was used for trapping in all patterning except in the adult stem cell co-culture system where a 100 × objective (Zeiss) with a NA 1.3 was used. A heated environmental chamber was constructed on top of the instrument to generate incubator-like conditions during patterning. A water based cooling system was constructed using coiled plastic tubing to reduce the temperature on the microscope stage for temperature dependent gelation applications.

### Cell Culture

The mES cell line (denoted CGR8) was cultured on 0.1% (v/v) gelatin solution. Medium consisted of Dulbecco's modified Eagle's medium (DMEM) (Life Technologies) supplemented with 10% (v/v) fetal bovine serum (FBS) (Sigma-Aldrich), 2 mM L-glutamine (Life Technologies), 0.1 μM 2-mercaptoethanol (Sigma-Aldrich), 50 μg/ml penicillin/streptomycin (Life Technologies) and 5,000 U/ml leukemia inhibitory factor (LIF) (Calbiochem). Cells were expanded, passaged and suspended in either avidin containing medium or pre-gel PEG solution ready for patterning on the HOTs system. Mouse MSCs (mMSCs) (Amsbio) were cultured according to manufacturer's guidelines and used for experimentation at passage four.

Primary mPCs were extracted from CD-1 neonatal mouse pups by serial digestion with Trypsin II S and collagenase I (Sigma-Aldrich). Growth medium consisted of alpha modified Eagle's medium with 10% (v/v) FBS (Sigma-Aldrich), 2 mM L-glutamine (Life Technologies), 0.1 μM 2-mercaptoethanol (Sigma-Aldrich) and 50 μg/ml penicillin/streptomycin (Life Technologies). All mPCs were used at passage three.

Immortalized human mesenchymal stem cells (ihMSCs) were generated by Lentiviral transfection of E6/E7 and hTERT genes as previously described^44^,^45^ using the Lentiviral vectors pLenti-hTERT, pLenti-III-SV40 and pLenti-III-HPV-16 E6/E7. The primary cells used for this purpose where commercially sourced human bone marrow mesenchymal stem cells which were initially expanded to passage three according to manufacturer's guidelines (Lonza).

### Polymer microfiber synthesis

Polymer electropsun fibers were produced using poly (lactic-co-glycolic acid) (PLGA) (75:25) (Lakeshore Biomaterials) as described previously^46^. These were then cryogenically ground by placing the fiber sheet into a pestle and mortar containing liquid N_2_ and ground for approximately 10 minutes. The resulting ground fibers were then fractioned according to size by passing through a series of sieves (Fisher Scientific) with fragments of <50 μm were used for patterning experiments. The fragments were functionalized for cell adhesion (Sigma-Aldrich) by suspension in 100 μg/ml fibronectin solution containing 50 μg/ml penicillin/streptomycin for 30 minutes at 37°C under constant agitation. The fibronectin solution was removed by centrifugation and fibers re-suspended in complete mES medium for 24 hours prior to patterning.

### Microparticle synthesis

Microparticles were fabricated using the high loading efficiency water-in-oil-in-water (w/o/w) emulsion method as previously described^33^. The microparticle batches were lyophilized and the powder was vacuum packed and stored at 4°C until required. The size distribution of microparticle batches was determined by suspension in deionized water (20 mg/ml) and sized using a laser diffraction method (Coulter LS230, Beckman Coulter, UK) (Supplementary Fig. 2). To test microparticle encapsulation efficiency microparticle samples were suspended in phosphate buffered saline (PBS) and placed under constant agitation (Gyrotwister, Fisher Scientific UK Ltd) at 37°C. The PBS was removed daily and assessed for Calcein AM content by means of absorbance (Calcein AM BLUE – 305 nm, Calcein AM Green – 284 nm) on a Nanodrop spectrophotometer (ND-1000, Labtech).

### Adivin-biotin mediated cell stabilization

The cellular organization during micromanipulation was in some cases stabilized by use of an avidin-biotin method as previously described^29^. Briefly, cells were passaged into suspension, washed twice with PBS and treated with 1 mM sodium periodate (Sigma-Aldrich) in the dark and at 4°C. The cells were then re-suspended in a 5 mM solution of biotin hydrazide solution in PBS with 0.1% FBS at a pH 6.5 for 30 minutes under constant rotational agitation. Finally, cells were re-suspended in relevant medium containing avidin at a concentration 100 μg/ml (Sigma-Aldrich).

### Hydrogel mediated cell and microparticle stabilization

The cellular organization during and after micromanipulation was in some cases stabilized by use of a PEG hydrogel system as previously described^30^. Pre-gel suspensions were cross-linked by addition of factor XIIIa (Fibrogammin, CSL Behring, Bern, Switzerland) either during optical tweezers based micromanipulation or immediately after. In other cultures a temperature dependent gelation protocol was employed by use of a 5% solution using ultra-low melting point (ULMP) agarose (Sigma-Aldrich) in PBS (Sigma-Aldrich) into which cells and polymer microparticles were suspended at 37°C. This temperature was maintained during micromanipulation and then lowered to approximately 20°C after patterning had been completed in order to induce *in situ* gelation.

### HOTs patterning procedures

All cell types (biotinylated and non-biotinylated) were suspended in avidin solution (10 μg/ml) PEG pre-gel solution, liquid agarose (<25°C) or an appropriate complete culture medium and placed into inbuilt microwells within polyHEMA coated glass bottomed culture dishes.

mES cells were positioned according to predetermined architectural arrangements using the holographic optical tweezers system either in mono-cultures or in conjunction with mMSCs and mPCs. Stabilization of the resulting constructs was achieved using the appropriate method (gels or avidin-biotin). All cell co-culture experiments were performed by generating mixed suspensions in avidin containing medium at a ratio of five mES to one mPC or mMSC. A green fluorescent cell tracker probe (CellTracker, Life Technologies) was used to label mPC cells and mMSCs for identification during co-culture experiments.

Polymer microparticles were positioned within or near to cellular constructs using the same procedures as the cells but were not functionalized with biotin in any experiments and were stabilized using ULMP agarose. Fibronectin coated PLGA fragments were diluted at a ratio of 1:10 and suspended in 10 ml of 100 cell/ml mES solution.

### Generation of adult stem cell co-culture system

Fragments of de-cellularized and demineralized trabecular bone were generated based on a previously described method^34^. These were then re-cellularized with mPCs by incubation in complete medium at a density of 1 × 10^6^ cells/ml and placed under continuous agitation for 12 hours. The remaining cell suspension was then removed and replaced with complete culture medium. The resulting re-cellularized fragments were left in culture for a further 24 hours before use with the HOTs patterning procedure. ihMSCs were passaged and suspended in a microwell containing complete medium and a single re-cellularized ECM fragment. HOTs were then used to position individual ihMSCs into specific locations within the re-cellularized ECM fragments and relative positions noted for later images. The fragments were then left at 37°C and 5% CO_2_ for 72 hours.

## Author Contributions

G.R.K. designed and performed experiments, analyzed data and wrote the manuscript and supplementary material. E.B., T.U. and J.W. designed and performed experiments and analyzed data. G.M.G. was the lead for the construction and design of optical tweezers instrument. Y.D. formulated and prepared hydrogel systems. M.E., M.P., S.A. and L.D.B. supplied supervisory input into experimental design and interpretation of data. K.S. was the project leader, supervised experimental design and interpretation of data and contributed to manuscript text. All authors discussed the results and implications of the project as well as commenting on the manuscript at all stages.

## Supplementary Material

Supplementary Information Supplementary Information

Supplementary Information Supplementary Movie 1

Supplementary Information Supplementary Movie 2
