# Supplementary material for: Precision Assembly of Complex Cellular Microenvironments using Holographic Optical Tweezers
Source: Sci Rep. 2015 Feb 26;5:8577. doi: 10.1038/srep08577 (PMC4341216; doi:10.1038/srep08577)
Supplement: Supplementary Information [file srep08577-s1.pdf]

Precision Assembly of Complex Cellular Microenvironments using Holographic Optical Tweezers

(supplementary information)

Glen R Kirkham[1], Emily Britchford[1], Thomas Upton[1], James Ware[1], Graham M Gibson[2], Yannick Devaud[3], Martin Ehrbar[3], Miles Padgett[2], Stephanie Allen[1], Lee D Buttery[1] and Kevin Shakesheff[1]

[1]School of Pharmacy, University of Nottingham, Nottingham, NG7 2RD, UK
[2]School of Physics and Astronomy, University of Glasgow, Glasgow, G12 8QQ, UK
[3]Department of Obstetrics, University Hospital Zurich, 8091 Zurich, Switzerland

Supplementary Figures

Supplementary Figure 1. A. Schematic of the holographic optical tweezers system. A 1064 nm laser is expanded to fill an SLM, displaying computer controlled holograms which are used to generate multiple trapping beams. The beams are coupled into the microscope using a polarizing beamsplitter enabling the generation of multiple optical traps in 3D within the sample. B. A schematic of the support systems installed on the microscope stage to maintain optimal cellular function during and after patterning as well as provide cooling for temperature based gelation protocols. C. A diagrammatic representation of the *in situ* use of an avidin-biotin method (I) and two hydrogel systems (II) used to stabilize cellular architectures after optical tweezers based micromanipulation.

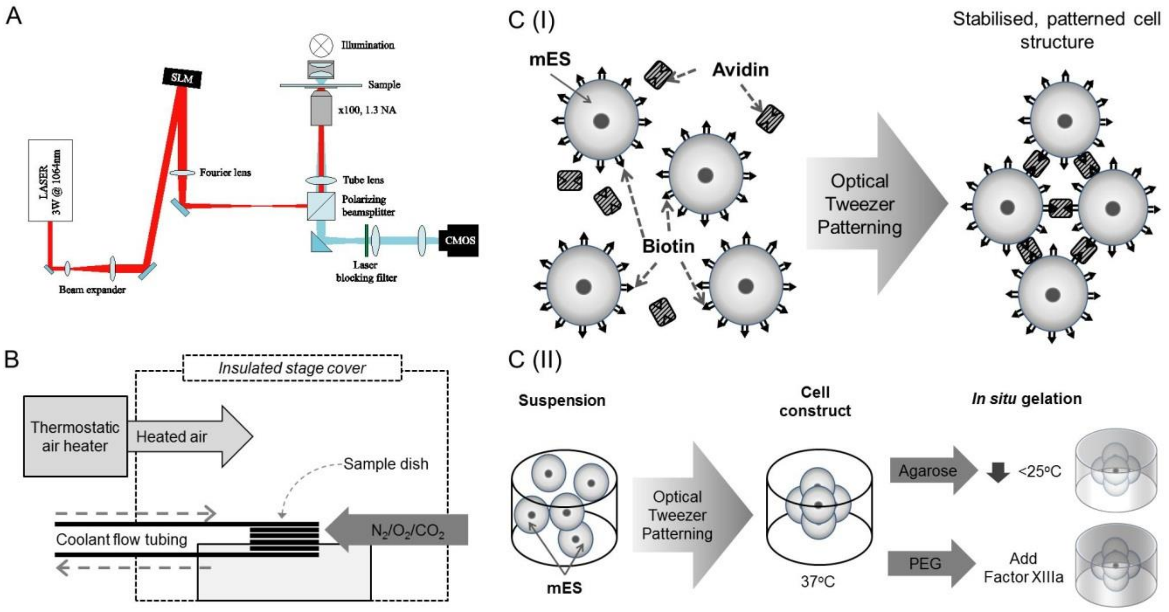

Supplementary Figure 2. PLGA polymer microparticle size distribution, determined by use of a Coulter counter for both blue (A) and green (B) Calcein-AM loaded batches.

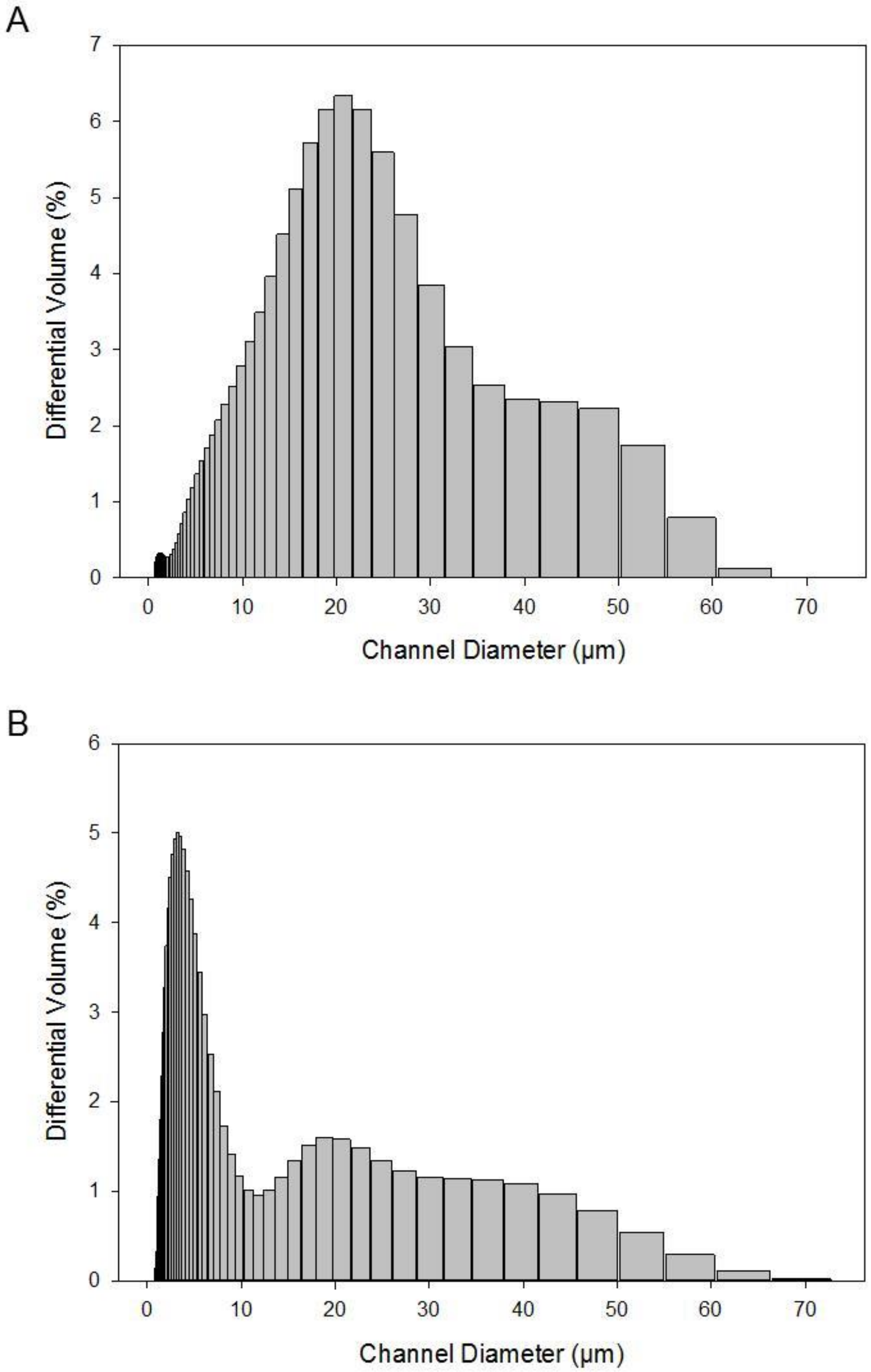

Supplementary Figure 3. Viability of mouse embryonic stem cells assessed using a fluorescent live/dead stain: A. After construction into six cell ring structures and exposure to holographic optical tweezers based trapping of 10, 20 and 30 minute durations. Scale bars = 12 µm. B. After construction into six cell ring structures but with no additional trapping exposures. Scale bars = 12µm. C. Cells not patterned and not exposed to any form of optical trapping or laser energies. Scale bars = 85µm. Images in A, B and C were taken 1 hour after patterning procedures.

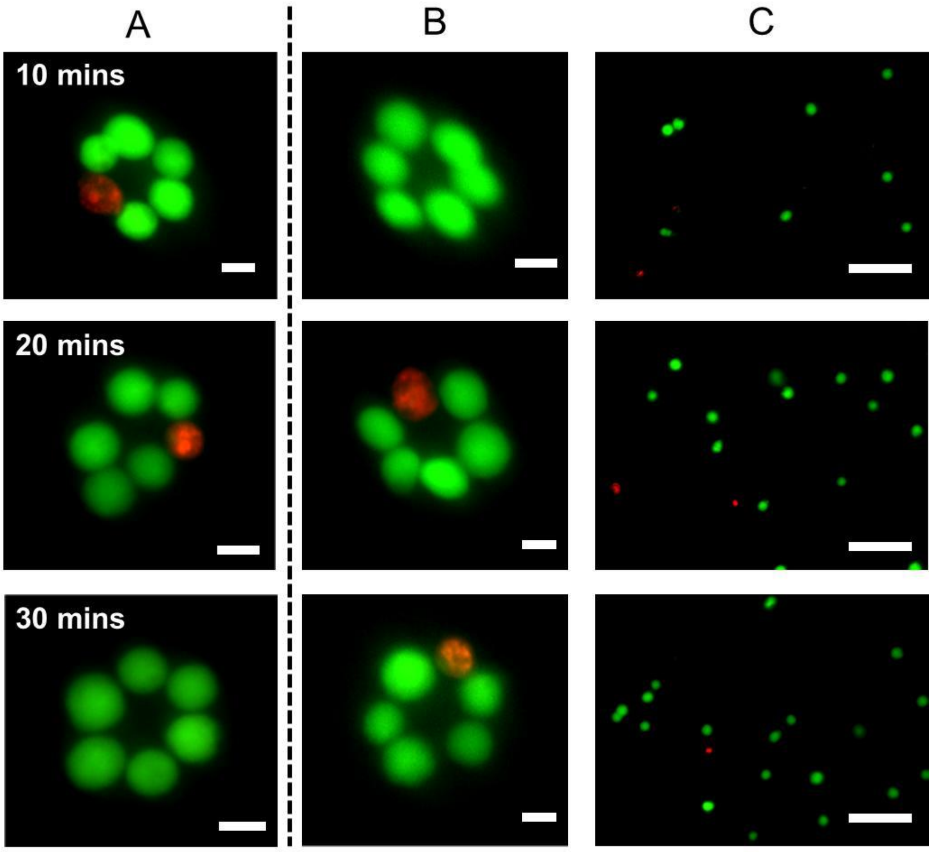

Supplementary Movie 1. Micromanipulation of mouse embryonic stem cells suspended in medium using a holographic optical tweezers micromanipulation technique. The movie shows three distinct patterning experiments that were sequentially integrated into a single avi file. The speed of this sequence was increased by a factor of three.

Supplementary Movie 2. A 3D reconstructed confocal microscope projection of 66 mouse embryonic stem cells positioned into a predetermined structure using a holographic optical tweezers micromanipulation technique. The cells were labeled with Calcein-AM green prior to pattering and the structure stabilized by the initial use of avidin-biotin and the subsequent gelation of a PEG based modular hydrogel.
